# A complex form of hereditary spastic paraplegia harboring a novel variant, p.W1515*, in the *SPG11* gene

**DOI:** 10.1016/j.ensci.2021.100391

**Published:** 2022-01-03

**Authors:** Kensuke Daida, Yosuke Nishioka, Yuanzhe Li, Hiroyo Yoshino, Manabu Funayama, Nobutaka Hattori, Kenya Nishioka

**Affiliations:** a Department of Neurology, Juntendo University School of Medicine, 2-1-1 Hongo, Bunkyo-ku, Tokyo 113-8421, Japan; b Nishioka Memorial Central Clinic, 375 Hasama, Isobecho, Shima-shi, Mie 517-0214, Japan; c Research Institute for Diseases of Old Age, Graduate School of Medicine, Juntendo University, 2-1-1 Hongo, Bunkyo-ku, Tokyo 113-8421, Japan

**Keywords:** Hereditary spastic paraplegia, Whole exome sequencing, MRI, SPG11, Ears of the lynx sign

## Abstract

Individuals with hereditary spastic paraplegia (HSP) are known to present with a variety of symptoms, including intellectual disability, cognitive decline, parkinsonism, and epilepsy. We report here our experience of treating a family with consanguinity, including three patients with HSP-related symptoms. We performed whole-exome sequencing and identified a novel pathogenic nonsense variant, c.4544G > A, p.W1515*, in the *SPG11* gene. Proband and her affected sister showed the same course of gait disturbance due to spastic paraplegia from childhood and progressive cognitive decline from early adulthood. Brain MRI depicted a thinning of the corpus callosum, severe atrophic changes in the frontotemporal lobes, and ears of the lynx sign. Patients with *SPG11* variants clinically present with distinctive symptoms.

## Highlights

• HSP type 11 is a rare clinical and genetic heterogeneous disorder.
• We present three cases in a family with a complex form of HSP type 11.
• We identified a novel nonsense variant, c.4544G > A, p.W1515*, in *SPG11.*
• Brain MRI is an important tool to help with the diagnosis of HSP type 11.

## 1 Introduction

Hereditary spastic paraplegia (HSP) is a rare and heterogenous neuro-degenerative disorder that presents with slowly progressive spasticity in the lower limbs. It has been clinically classified into the following two types: (i) pure HSP, which is mainly characterized by spastic paraplegia, and (ii) complex HSP, which is characterized by spastic paraplegia and other neurological symptoms (such as ataxia, intellectual disabilities, and slowly progressing cognitive decline) and non-neurological symptoms (such as ophthalmological abnormalities, dysmorphic features, and orthopedic abnormalities) [1]. The *SPG11 vesicle trafficking associated, spatacsin* (*SPG11*) gene is the most common pathogenic gene in autosomal recessive families [2]. The *SPG11 gene* is located on chromosome 15q21.1 and includes 40 exons. Patients with HSP type 11 (SPG11-HSP) have been reported to initially show progressive spastic paraplegia followed by additional symptoms such as cognitive decline, parkinsonism, psychosis, visual impairments, polyneuropathy, sphincter disturbance, and symptoms related to upper and lower motor neurons that resemble those of slowly progressive amyotrophic lateral sclerosis [3].

Herein, we present the case of a Japanese family in which three patients presented with a complex HSP, in whom we identified a novel homozygous variant in the *SPG11* gene.

## 2 Material and methods

This study was approved by the ethics review committee of the Juntendo University School of Medicine. We enrolled four family members [father (III-1), mother (III-4), proband (IV-2), and affected sister (IV-3)] to our genetic test. We obtained informed consent from the parents (III-1, III-4), not from two affected sisters (IV-2, IV-3) due to the cognitive decline. Genomic DNA was extracted by the standard method from the family members (III-1, III-4, IV-2, and IV-3). We implemented whole-exome sequencing of 150 bp paired-end sequencing on NovaSeq6000 (Illumina, San Diego, CA, USA). Sample preparation for whole exome sequencing was performed using the SureSelect Human All Exon V6 Kit (Agilent Technologies, Santa Clara, CA, USA). We referred read alignment to human genome (UCSC GRch38/hg38) with Burrows-Wheeler Aligner version 0.7.17-r1188. Single nucleotide variants were detected in each patient using SAMtools version1.10. Variant calling, indel realignment, and base quality score recalibration were performed with GATK v.4.1.3.0. The variants identified by next-generation sequencing were filtered according to the following criteria: location in exons or splice sites; the allele frequency in public databases (gnomAD) smaller than 0.001 [4], being carried in the homozygous state in two affected sisters (IV-2, IV-3), heterozygous state in mother and father (III-1, III-4); prediction of being non-synonymous or cause aberrant splicing. All the identified variants were confirmed by Sanger method.

## 3 Results

### 3.1 Case report

**Fig. 1:**
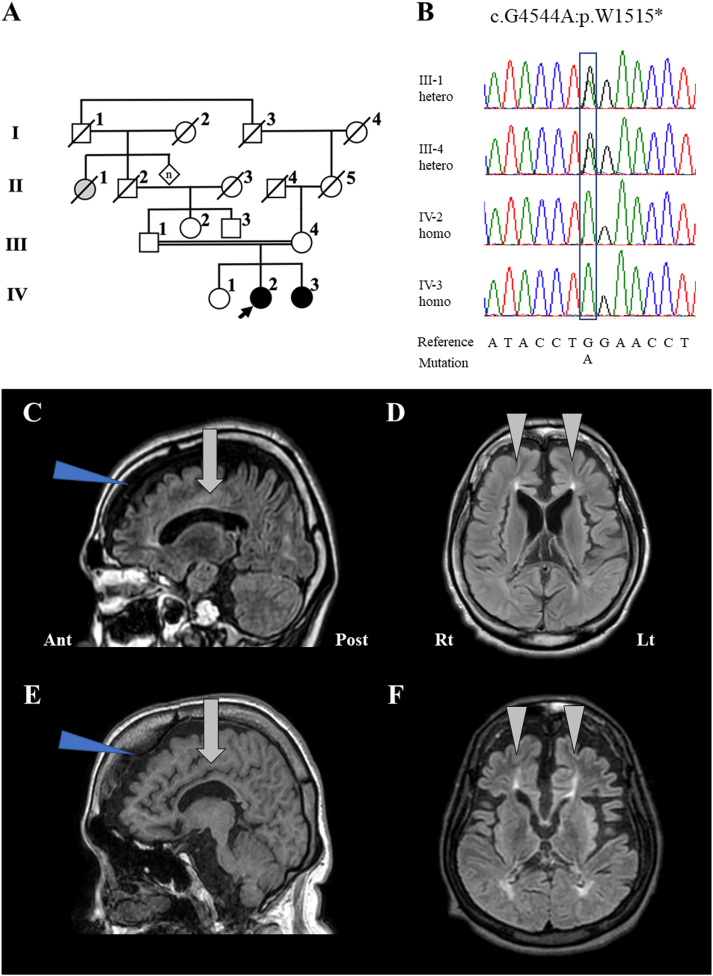
Family pedigree, brain MRI, and Sanger sequencing of family members. (A) The symbols indicate the following: square, man; circle, woman; white colored, asymptomatic individual; black colored, symptomatic individual with a variant p.W1515* in the *SPG11* gene; gray colored, symptomatic individual without genetic test; double line, consanguinity; black arrow, a proband; oblique line, deceased individual. (B) The results of Sanger sequencing present the homozygous variants, proband (IV-2) and affected sister (IV-3), and heterozygous variants, father (III-1) and mother (III-4), of c.4544G > A, p.W1515*. (C) to (F) Brain MRI with fluid-attenuated inversion recovery. (C) and (D): proband (IV-2). (E) and (F): affected sister (IV-3). Severe atrophic changes in the frontal lobes (*blue triangle*). The thinning of corpus collosum (*gray arrow*). Ears of lynx sign (*gray triangle*). Abbreviations: homo, homozygote; hetero, heterozygote; Ant, anterior; Post, posterior; Rt, right; Lt, left. (For interpretation of the references to colour in this figure legend, the reader is referred to the web version of this article.)

The patients lived in the small village located in the western area from Tokyo. It is about a consanguineous family in which two sisters (IV-1 and IV-4) presented with symptoms consistent with complex HSP (Fig. 1A). Their paternal grand-aunt (II-1) also presented a medical history of spastic paraplegia.

### 3.2 Case 1: the proband (IV-2)

She had a normal developmental history from infancy through childhood. However, her mother noticed nystagmoid movements during her childhood, and she had toe walking since she was in junior high school. She experienced difficulties in both academics and physical training. She graduated from high school and worked at a store for 2 years. At 20 years of age, she exhibited continuous slow movements, and her gait disturbance progressed. From the age of 30 years, she was unable to stand by herself and used a wheelchair, and her cognitive dysfunction slowly progressed. After this, she was admitted to the nursing facility. At 41 years of age, our first examinations revealed severe cognitive dysfunction, gait disturbance, disuse atrophy in all limbs, severe spasticity, hyper-tonus, contracture, and pes equinus in the lower limbs. She was only able to speak simple words such as “Ah” or “Uh,” and displayed a smile and euphoric expression in response to someone. Deep tendon reflexes showed hyperreflexia in the upper limbs. Pathological reflexes, such as palmomental, Hoffman, and Tromner reflexes were observed. Forced grasping was seen. She was bed ridden for most of the day. Her brain magnetic resonance imaging (MRI) showed atrophic changes in the frontal lobe, thinning of the corpus callosum (Fig. 1C), and ears of the lynx sign in the frontal horn of the lateral ventricle (Fig. 1D).

### 3.3 Case 2: the younger sister of the proband (IV-3)

She graduated from high school without major problems. Her school grade was not good. However, she normally communicated with others without character changes, forgetfulness, or cognitive decline. After graduation, she could work well at a company. At 23 years of age, she noticed difficulties while walking, and was unable to fix the lower limbs in a standing posture. At 27 years of age, she experienced severe infection and was admitted to another hospital. Following this, her gait disturbance progressed. At the age of 38 years, our examinations found that she was bed-ridden all day and was unable to communicate with others; she presented with roving eye movements, forced laugh, decorticate posture, disuse atrophies, contracture, and pes equinus in the extremities. Deep tendon reflexes were absent due to severe contracture. She was administered fluid diets and medications via the gastric fistula. Brain MRI revealed findings similar to the proband (IV-2) (Fig. 1E, F).

### 3.4 Case 3: grandaunt of the proband (II-1)

There was another patient in the family (II-1) without our genetic test. Her history at infancy remained unclear. During her childhood, she was diagnosed with intellectual disability and used a wheelchair due to gait disturbance. She dropped out of the primary school and lived in the nursing facility in her later years. She died at 90 years of age due to gastrointestinal hemorrhage. Her parents (I-1 and I-2) did not show any symptoms related to neurological disorders.

### 3.5 Genetic tests

Through whole-exome sequencing, we selected two homozygous variants; *SPG11*: NM_001160227 c.4544G > A: p.W1515*, and *EP300 interacting inhibitor of differentiation 1* (*EID1*): NM_014335:exon1:c.337G > C: p.E113Q. The variant of the *SPG11* gene, which is known to cause hereditary spastic paraplegia, was so rare that it had not been recorded in the “Genome Medical Alliance Japan, Whole Genome Aggregation (GEM-J WGA) panel” by Genome Medical alliance Japan Project (GEM-J) (https://togovar.biosciencedbc.jp/doc/datasets/gem_j_wga), and Tohoku Medical Megabank Organization (ToMMo 4.7KJPN) (https://jmorp.megabank.tohoku.ac.jp/202008/). Conversely, the allele frequency of the variant of *EID1*, which, to our knowledge, has not been reported to be a cause of HSP, is 0.002 in GEM-J WGA (16/9546) and ToMMo 4.7KJPN (25/15172). Therefore, we considered that the stop codon variant p.W1515* of the *SPG11* gene would be the presumed pathogenic variant in the family; this identified variant, p.W1515*, was confirmed by Sanger sequencing (Fig. 1B).

## 4 Discussion

We identified a nonsense variant in the *SPG11* gene from a family with complex HSP and autosomal recessive inheritance. To our knowledge, there have been no reports of c.4544G > A, p.W1515* in the *SPG11* gene. Whole exome sequencing is a utility tool to identify the pathogenic genes related to HSP, due to many related genes reported previously (over 70) to HSP. Up to date, several types of variants in the *SPG11* gene have been reported as a causative factor such as frameshift, missense, nonsense, or splice-site variant [5]. The patients of HSP with nonsense variants in the *SPG11* gene showed onset at their 10's, spasticity, and weakness in the lower limbs with extensor plantar responses, dysarthria, intellectual disability, and cognitive decline [6,7]. Mutations of the *SPG11* gene have been reported for other phenotypes, namely, Charcot-Marie-Tooth disease and amyotrophic lateral sclerosis [8,9]. To our knowledge, reported pathogenic mutations in exon 26 of the *SPG11* gene, where p.W1515* is positioned, are all related to HSP. However, the position of the mutation does not seem to be clearly related to the phenotype [9]. It might be still premature to describe the SPG11-HSP phenotype based on the localization of the mutation.

The brain MRI findings helped us to establish a robust clinical diagnosis of SPG11-HSP according to three characteristic findings, as follows: (i) a thin corpus callosum, (ii) atrophic changes in the frontotemporal lobes, and (iii) the ears of the lynx sign in the anterior horn of the lateral ventricles (Table 1 and Supplementary data) [6,7]. The Ears of the lynx sign has been frequently observed finding in patients with pathogenic variants in the *SPG11* gene and *ZFYVE26* (SPG15), which presents as hyperintensity in fluid-attenuated inversion recovery, and hypointensity in T1-weighted images of the forceps minor of the corpus callosum or the anterior horn of the lateral ventricles [10]. The high sensitivity and specificity of the ears of the lynx sign (78.8–97.0%, and 90.9–100%, respectively) meant that other neurological disorders could be excluded [10]. A finding of the thin corpus callosum is known to be a major phenotypic hallmark of patients with autosomal recessive HSP, especially that involving the *SPG11* gene and *ZFYVE26* [3]. The combined findings of both a thin corpus callosum and the ears of the lynx sign might be an effective indicator to increase the sensitivity and specificity of neuroimaging-based diagnosis of the patients with pathogenic variants in the *SPG11* gene or *ZFYVE26*.

**Table 1:** Differences of MRI findings of the patients with complex HSP.

Genotype of SPG	MRI findings	Location	Notes	References
SPG2	Diffuse pattern of hypomyelination T2weighted hyperintensities	Spine	Atrophy was more severe in SPG6 and SPG8	Hedera et al. (2005) [s11]
SPG7 (i)	Cerebellar atrophy	Brain	Frequency: 39–95%.	van Gassan et al. (2012) [s12], Hewamadduma et al. (2018) [s13].
SPG7 (ii)	Iso- or hypontensities in the dentate nuculeus, pontine, and white matter in T2-WI	Brain	Frequency: 86%	Hewamadduma et al. (2018) [s13].
SPG11/SPG15	Thin of the corpus callosum and hyperintensities in the periventricular white matter, and ears of the Lynx sign.	Brain	Ears of the Lynx sign: a specific pattern of T2weighted hyperintensities at the anterior forceps of the corpus callosum	Pensato et al. (2014) [5], Kara et al. (2016) [s14], Pascual et al. (2019) [10].
SPG35	White matter changes, hypointensity of the globus pallidus, ponto-cerebellar atrophy, and thin of corpus callosum.	Brain	Three of four imaging features are present in 85% of FA2H mutation carriers	Rattay et al. (2019) [s16]

Abbreviation: SPG, spastic paraplegia; MRI, magnetic resonance imaging. References [s11-15] were described in the supplementary data.

In conclusion, we identified a novel variant of the *SPG11* gene in a family with complex HSP. Our study confirms the clinical and genetic heterogeneity of SPG11.

## Appendix A Supplementary data

Supplementary material Image 1
