# Supplementary material for: A complex form of hereditary spastic paraplegia harboring a novel variant, p.W1515*, in the SPG11 gene
Source: eNeurologicalSci. 2022 Jan 3;26:100391. doi: 10.1016/j.ensci.2021.100391 (PMC8749458; doi:10.1016/j.ensci.2021.100391)
Supplement: Supplementary file 1 — Supplementary material [file mmc1.docx]

**Supplementary data**

**References in the Table 1**

[5] V. Pensato, B. Castellotti, C. Gellera, D. Pareyson, C. Ciano, L. Nanetti, E. Salsano, G. Piscosquito, E. Sarto, M. Eoli, I. Moroni, P. Soliveri, E. Lamperti, L. Chiapparini, D. Di Bella, F. Taroni, C. Mariotti, Overlapping phenotypes in complex spastic paraplegias SPG11, SPG15, SPG35 and SPG48, Brain : a journal of neurology 137(Pt 7) (2014) 1907-20.

[10] B. Pascual, S.T. de Bot, M.R. Daniels, M.C. França, Jr., C. Toro, M. Riverol, P. Hedera, M.T. Bassi, N. Bresolin, B.P. van de Warrenburg, B. Kremer, J. Nicolai, P. Charles, J. Xu, S. Singh, N.J. Patronas, S.H. Fung, M.D. Gregory, J.C. Masdeu, "Ears of the Lynx" MRI Sign Is Associated with SPG11 and SPG15 Hereditary Spastic Paraplegia, AJNR. American journal of neuroradiology 40(1) (2019) 199-203.

[s11] P. Hedera, O.P. Eldevik, P. Maly, S. Rainier, J.K. Fink, Spinal cord magnetic resonance imaging in autosomal dominant hereditary spastic paraplegia, Neuroradiology 47(10) (2005) 730-4.

[s12] K.L. van Gassen, C.D. van der Heijden, S.T. de Bot, W.F. den Dunnen, L.H. van den Berg, C.C. Verschuuren-Bemelmans, H.P. Kremer, J.H. Veldink, E.J. Kamsteeg, H. Scheffer, B.P. van de Warrenburg, Genotype-phenotype correlations in spastic paraplegia type 7: a study in a large Dutch cohort, Brain : a journal of neurology 135(Pt 10) (2012) 2994-3004.

[s13] C.A. Hewamadduma, N. Hoggard, R. O'Malley, M.K. Robinson, N.J. Beauchamp, R. Segamogaite, J. Martindale, T. Rodgers, G. Rao, P. Sarrigiannis, P. Shanmugarajah, P. Zis, B. Sharrack, C.J. McDermott, P.J. Shaw, M. Hadjivassiliou, Novel genotype-phenotype and MRI correlations in a large cohort of patients with SPG7 mutations, Neurology. Genetics 4(6) (2018) e279.

[s14] E. Kara, A. Tucci, C. Manzoni, D.S. Lynch, M. Elpidorou, C. Bettencourt, V. Chelban, A. Manole, S.A. Hamed, N.A. Haridy, M. Federoff, E. Preza, D. Hughes, A. Pittman, Z. Jaunmuktane, S. Brandner, G. Xiromerisiou, S. Wiethoff, L. Schottlaender, C. Proukakis, H. Morris, T. Warner, K.P. Bhatia, L.V. Korlipara, A.B. Singleton, J. Hardy, N.W. Wood, P.A. Lewis, H. Houlden, Genetic and phenotypic characterization of complex hereditary spastic paraplegia, Brain : a journal of neurology 139(Pt 7) (2016) 1904-18.

[s15] T.W. Rattay, T. Lindig, J. Baets, K. Smets, T. Deconinck, A.S. Söhn, K. Hörtnagel, K.N. Eckstein, S. Wiethoff, J. Reichbauer, M. Döbler-Neumann, I. Krägeloh-Mann, M. Auer-Grumbach, B. Plecko, A. Münchau, B. Wilken, M. Janauschek, A.K. Giese, J.L. De Bleecker, E. Ortibus, M. Debyser, A. Lopez de Munain, A. Pujol, M.T. Bassi, M.G. D'Angelo, P. De Jonghe, S. Züchner, P. Bauer, L. Schöls, R. Schüle, FAHN/SPG35: a narrow phenotypic spectrum across disease classifications, Brain : a journal of neurology 142(6) (2019) 1561-1572.
